# Type-1 spinal muscular atrophy cohort before and after disease-modifying therapies

**DOI:** 10.1055/s-0044-1791757

**Published:** 2024-11-06

**Authors:** Brenda Klemm Arci Mattos de Freitas Alves, Alexandra Prufer de Queiroz Campos Araujo, Flávia Nardes dos Santos, Márcia Gonçalves Ribeiro

**Affiliations:** 1 Universidade Federal do Rio de Janeiro, Pós-graduação em Saúde Materno-infantil, Rio de Janeiro RJ, Brazil.; 2 Universidade Federal do Rio de Janeiro, Faculdade de Medicina, Departamento de Pediatria, Rio de Janeiro RJ, Brazil.

**Keywords:** Survival of Motor Neuron 1 Protein, Muscular Atrophy, Spinal, Genetic Therapy, Proteína 1 de Sobrevivência do Neurônio Motor, Atrofia Muscular Espinhal, Terapia Genética

## Abstract

**Background** Spinal muscular atrophy (SMA-5q) is a neurodegenerative disease characterized by progressive muscle atrophy, hypotonia, and weakness, with SMA 1 presenting symptoms within the first 6 months of life. Disease-modifying therapies have been approved, with better outcomes with earlier treatment.

**Objective** To describe the safety and clinical efficacy of disease-modifying therapies based on *SMN1* and *SMN2* gene strategies concerning motor, respiratory, and bulbar function. Patients with SMA 1 were divided into 2 groups: those exclusively on nusinersen (group 1) and those transitioning to onasemnogene abeparvovec (OA) (group 2).

**Methods** Over 18 months, patients were assessed using the Children's Hospital of Philadelphia Infant Test of Neuromuscular Disorders (CHOP-INTEND) scale, developmental milestones, ventilation needs and duration, nutritional support needs, consistency of food, and signs of dysphagia. There were ten patients, divided between the groups; in group 1, the average age for starting nusinersen was 53.6 (12–115) months, and, in group 2, the age was 7 (1–12) months for nusinersen and 15.2 (10–19) months for OA.

**Results** Our results indicate that 70% of patients reached some motor milestones, with group 1 increasing by 10.2 points on the CHOP-INTEND scale, while group 2 increased by 33 points. Additionally, 90% of the patients experienced no respiratory decline, and 30% maintained oral feeding. No serious adverse effects or deaths were recorded.

**Conclusion** Both groups showed improvement in motor function and stabilization of respiratory and bulbar function, with the difference between the groups possibly being related to the earlier treatment initiation. Thus, the present study provides valuable insights into the real-world safety and clinical efficacy of disease-modifying therapies for SMA 1 patients.

## Resumo

**Antecedentes** A atrofia muscular espinhal (AME-5q) é uma doença neurodegenerativa caracterizada por atrofia muscular progressiva, hipotonia e fraqueza. Na AME 1 os sintomas iniciam-se no primeiro semestre de vida. Terapias modificadoras de doença foram aprovadas e demonstram melhores resultados quanto mais cedo forem iniciadas.

**Objetivo** Descrever a segurança e a eficácia clínica das terapias modificadoras de doença quanto às funções motora, respiratória e bulbar. Os pacientes com AME 1 foram divididos em 2 grupos: os que faziam uso exclusivamente de nusinersena (grupo 1) e os que transacionaram para onasemnogene abeparvovec (OA) (grupo 2).

**Métodos** Durante 18 meses, os pacientes foram avaliados utilizando a escala *Children's Hospital of Philadelphia Infant Test of Neuromuscular Disorders* (CHOP-INTEND), marcos do desenvolvimento, necessidade e duração da ventilação, necessidade de suporte nutricional, consistência dos alimentos e sinais de disfagia. Dividiram-se dez pacientes entre os grupos; no grupo 1, a idade média para início do nusinersena foi de 53,6 (12–115) meses e, no grupo 2, a idade foi de 7 (1–12) meses para o nusinersena e 15,2 (10–19) meses para OA.

**Resultado** Nossos resultados indicam que 70% dos pacientes atingiram algum marco motor, com o grupo 1 aumentando 10,2 pontos na escala CHOP-INTEND, e o grupo 2 aumentando 33. Ademais, 90% dos pacientes não apresentaram declínio respiratório e 30% mantiveram alimentação oral. Não houve efeitos adversos graves ou mortes.

**Conclusão** Ambos os grupos apresentaram melhoria na função motora e estabilização da função respiratória e bulbar, com a diferença entre os grupos sendo possivelmente relacionada com o início mais precoce do tratamento. Assim, este estudo fornece informações valiosas sobre a segurança e eficácia clínica destas terapias para pacientes com AME 1.

## INTRODUCTION

Spinal muscular atrophy linked to chromosome 5 (SMA-5q) is a neurodegenerative disease of autosomal recessive inheritance characterized by progressive muscle atrophy, hypotonia, and weakness, due to continuous degeneration of the α motor neurons of the spinal cord and brainstem. Its overall incidence is estimated at 1/10,000 live births, and despite being included in the group of rare diseases, it causes an important family, social, and economic impact, as it is one of the most common autosomal recessive hereditary disorders, and it is the monogenic disease with higher infant mortality. 1 2

Patients with SMA-5q have insufficient SMN protein (survival motor neuron protein), whose functions influence the axonal transport of molecules, mitochondrial metabolism, and ribonucleic acid (RNA) processing in neurons. The SMN protein is encoded by 2 genes, *SMN1* and its homologous gene, *SMN2* , located on chromosome 5. 3 4 5

In 96% of patients, SMA-5q is caused by a homozygous deletion (maternal and paternal alleles) of exons 7 and 8 of the *SMN1* gene, or, in some cases, only exon 7. Most patients (98%) inherit the mutated allele of both parents; in 2% of cases, a de novo mutation is seen in one of the alleles. The disease is diagnosed when genetic testing, by multiplex ligation-dependent probe amplification (MLPA) techniques or new generation sequencing, identifies either exon 7 or 8 deletions in both alleles or deletion in 1 allele and point mutations in the other allele of the *SMN1* gene. 6 7 8 9 10

The *SMN2* gene differs from *SMN1* by a single nucleotide variant (840C → T) in exon 7. This critical difference results in the exclusion of exon 7 from the majority (90%) of the transcripts during the processing of messenger RNA, resulting in the translation of a truncated and unstable SMN protein. Consequently, the *SMN2* gene can generate only 10% of functioning SMN protein. 3 4 5 Mutations in the *SMN1* gene cause the disease, and the *SMN2* gene acts as a phenotype modifier, that is, the greater the number of copies of *SMN2* genes, the less severe the clinical phenotype is, thus dividing the disease into 5 types according to the onset of symptoms and motor milestone achieved. 11

In SMA type 1, patients begin their symptoms between 0 and 6 months and never achieve sitting without support. There are also 3 subtypes: 1A in which symptoms appear before the 1 ^st^ month; 1B, between 1 and 3 months; and 1C, between 3 and 6 months. Eighty percent of patients with SMA type 1 have up to 2 copies of *SMN2* . 12

Currently, there are some disease-modifying therapies available, including gene therapy with the replacement of the *SMN1* gene (onasemnogene abeparvovec [OA]) and the inclusion of exon 7 in *SMN2* (nusinersen, risdiplan). 12 These therapies have already received approval from the main international regulatory agencies (Food and Drug Administration [FDA], European Medicines Agency [EMA]) and the Brazilian Health Regulatory Agency (Agência Nacional de Vigilância Sanitária [ANVISA], in Portuguese). 13 14 Clinical trials have demonstrated improved survival, respiratory function, muscle strength, and gains in motor milestones, in a magnitude that is proportional to the earliness of treatment. 15 16 17 18 19 20

The present study aims to evaluate the safety and clinical efficacy of disease-modifying therapies based on *SMN1* and *SMN2* gene strategies, in particular, nusinersen and OA, over an 18-month follow-up of a cohort of patients with type-1 SMA concerning motor, respiratory, and bulbar function, ratifying the importance of including these drugs in the public health system.

## METHODS

The current observational and retrospective study involved 10 patients with SMA 5q type 1, aged 1 to 130 months, and followed up between 2018 and 2023. These patients belong to the center for neuromuscular diseases, with a database that monitored more than 50 patients with SMA since 1989. The inclusion criteria were patients with SMA 5q type 1, with genetic diagnosis confirmation, who used nusinersen and/or OA between 2018 and 2023. The exclusion criteria were patients who did not maintain follow-up over 18 months and patients with cerebral hypoxia due to asphyxia or sepsis, given the risk of central motor disabilities affecting the analysis of results. This study was approved by the institution's Ethics and Research Committee (approval number 5.495.007), and all guardians of participants signed an informed consent form. Nusinersen was applied in our hospital or in a private hospital, and OA was delivered in hospitals outside the state. In our center, all patients follow standard care recommendations. 21 22 23

Clinical and demographic data were collected before and over 18 months after disease-modifying therapies. The numerical variables were the ages at symptom onset, non-invasive or invasive ventilation, gastrostomy or nutritional support, developmental milestones, onset of nusinersen, and gene therapy as well as Children's Hospital of Philadelphia Infant Test of Neuromuscular Disorders (CHOP INTEND) score, 24 *SMN2* copy number, and daily ventilation time. The categorical variables were: SMA-1 subtype, type of mutation, type of ventilatory support (invasive or noninvasive), adverse effects, feeding route, food consistency, and signs of dysphagia, such as coughing and choking. Indirect clinical variables of respiratory function were used, such as ventilatory support and daily ventilation time, since young children are unable to cooperate with peak flow measurements and spirometry, and we did not have access to capnography.

The patients were divided into 2 groups: group 1 patients who used only nusinersen (50%), and group 2, patients who switched to OA from nusinersen (50%). Patients obtained disease-modifying therapies through the Brazilian public health system (Sistema Único de Saúde [SUS], in Portuguese), health insurance, or legal proceedings. Before patients in group 2 received OA, nusinersen was suspended and was not returned to afterward.

Prescriptions in our service are only those approved and available in public health care (SUS). However, patients have access to information through social media and seek simultaneous monitoring with external professionals, who have a different approach to that used in our service and to that proposed by specialists. 23

Trained physicians fill out the center's standardized clinical assessment forms at consultations. The same physiotherapist carries out the motor scale assessment.

Statistical analysis was performed using the Microsoft Excel software (Microsoft Corp., Redmond, WA, USA) for Mac version 16.8 with the distribution of frequencies and measures of central tendency and dispersion.

## RESULTS

Ten out of 18 patients with SMA type 1 were included in the present study, 4 with type 1B and 6 with type 1C. Two had homozygous deletion of exon 7, 7 had homozygous deletion of exon 7 and 8, and 1 patient had compound heterozygosity. Eight had 2 copies of *SMN2* , 1 had 3 copies, and 1 had no information about the number of copies. This last patient presented with hypotonia at 3 months and at the age of 6 months was diagnosed with SMA after genetic testing (polymerase chain reaction [PCR]), but she did not have access to the MLPA test.

The mean age at symptom onset was 2.8 months (1–5). In group 1, the mean age for starting nusinersen was 53.6 months (12–115), and in group 2, the mean age for nusinersen was 7 months (1–12), and for OA, it was 15.2 months (10–19). However, one of the patients who used both medications was presymptomatic when she started the first drug, at 1 month of age (and began to show symptoms at 1.5 months of age). All patients in group 2 underwent OA prescribed and administered by teams from other hospitals, even though no worsening was observed on nusinersen.

No patient presented adverse effects related to nusinersen. Among the patients who received OA, 1 presented changes in liver enzymes after 4 months and needed to restart prednisolone, with improvement. No patients died.

### Motor assessment

**Figure 1:**
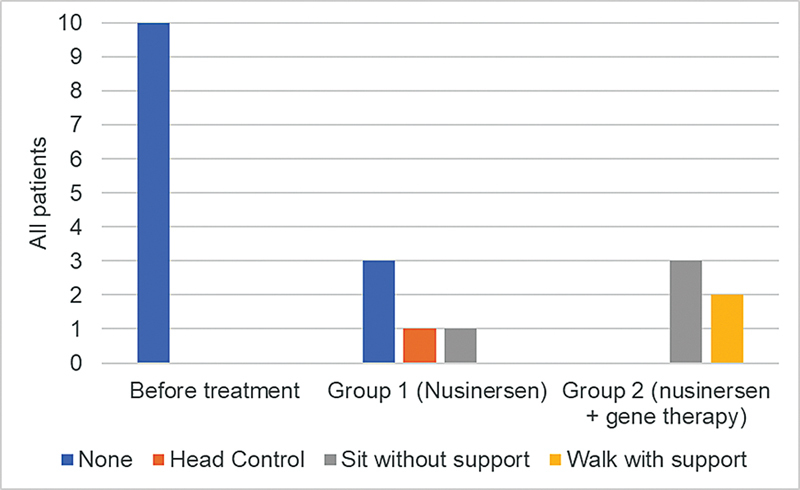
Achieved motor milestones before treatment and after 18 months of treatment in groups 1 and 2.

Before the treatment, none of the patients had head control or any other motor milestone. In group 1, with patients who only took nusinersen (5), 3 did not reach any motor milestones, 1 managed to have head control at 43 months, and 1 was able to sit without support at 21 months. Among the patients who started earlier and switched medication (5), 3 were able to sit without support (average of 34 months), and 2 of them walked with support (average of 23.5 months) ( Figure 1 ).

At baseline, the score on the CHOP-INTEND scale was between 3 and 41 (average of 18.3). After 18 months, scores were between 6 and 64 (average of 39.9), with a mean increase of 21.6 points. An average increase of 10.2 points was noted in group 1, whereas in group 2, this increase reached 33 points.

Seventy percent of patients in this study achieved at least one motor milestone, with two patients being able to walk with support, and all patients who underwent gene therapy achieved a CHOP INTEND of at least 48.

### Respiratory support

Ninety percent of the patients had no worsening of respiratory function. No patient developed the need for invasive ventilation throughout the evaluation. Only one patient had an increase in the number of hours of daily ventilation above 16 h/day, but this was a preventive rather than therapeutic measure.

**Figure 2:**
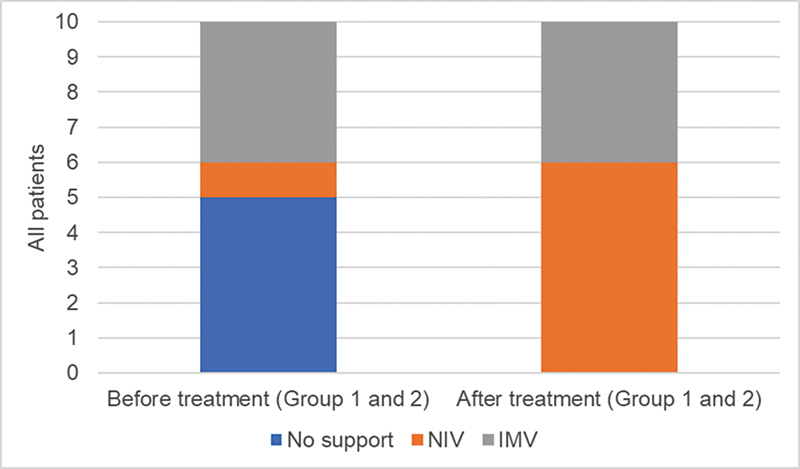
Abbreviations: NIV, noninvasive ventilation; IMV, invasive mechanical ventilation with tracheostomy. Respiratory support before treatment and after 18 months of treatment in groups 1 and 2.

At baseline, four patients were using invasive ventilation via tracheostomy, two patients were using non-invasive ventilation, and four did not need ventilation. In the end, the patients who were using invasive ventilation remained that way, and the other six patients were using non-invasive ventilation ( Figure 2 ).

Regarding daily ventilation time, among patients with tracheostomy, 3 maintained time greater than 20 h/day, and 1 patient at baseline used it for 13 h, and, at the end, he did it for 16 h. Among patients who completed the evaluation with non-invasive ventilation (6/10), only 1 exceeded the use of 16 h/day.

Although all patients were on ventilatory support at the end of 18 months, we were able to observe that there was no progression to tracheostomy and only 1 of the patients increased her daily ventilation time to over 16 h/day.

### Bulbar function

**Figure 3:**
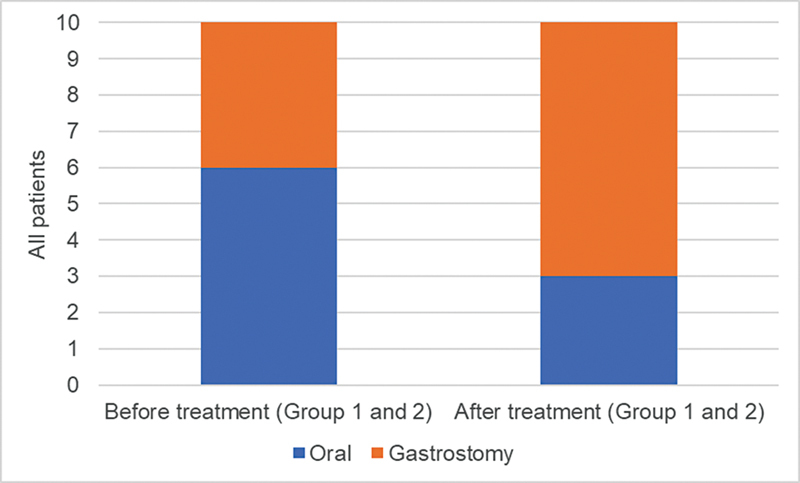
Feeding route before treatment and after 18 months of treatment in groups 1 and 2.

The improvement in bulbar function was very variable among the patients. In the beginning, four patients used a gastrostomy. At the end of the assessment, seven patients used the gastrostomy as a feeding route and three were fed orally ( Figure 3 ). The patients who progressed to gastrostomy had signs of dysphagia (coughing and choking).

Of the patients who maintained the oral route, two were able to eat solid food and one was able to eat soft food. One patient was able to speak meaningful sentences.

## DISCUSSION

These clinical advances represent notable deviations from the findings of studies on the natural history of the disease, which report a progressive drop in CHOP INTEND over time and failure to reach the motor milestone of sitting without support. Additionally, in these studies, the average age of death or permanent ventilation is below 2 years, with a 50% chance of reaching this at 12 months. 25 26 27 28

**Figure 4:**
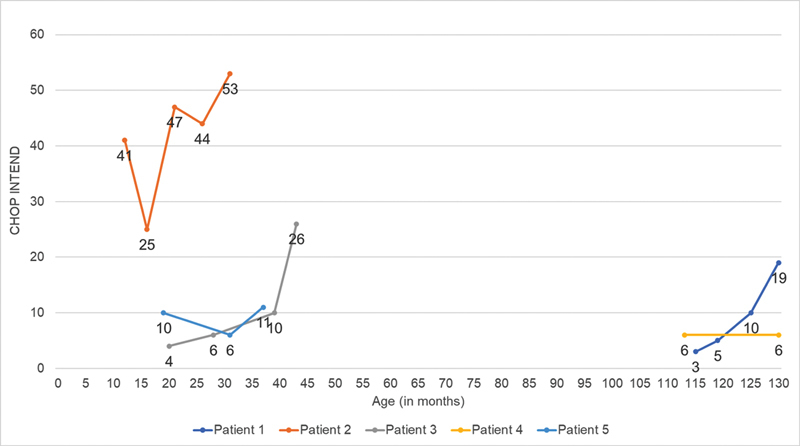
Evolution of the score on the Children's Hospital of Philadelphia Infant Test of Neuromuscular Disorders scale in patients who received only nusinersen (group 1).

Unlike clinical trials with nusinersen, 16 29 80% of our patients in group 1 started treatment after more than 12 months of illness and required permanent ventilation with a tracheostomy. ( Table 1 ) Only one patient in this group started treatment earlier and obtained the highest CHOP INTEND score ( Figure 4 ), managing to sit up without support and maintaining oral feeding with soft foods. Even in such a heterogeneous group, it was possible to observe improvements in the CHOP INTEND scale, gains in motor milestones in two patients, and stability in bulbar and ventilatory function. No one had any side effects. This response is in accordance with the literature and the statement “time is motor neuron” 30 .

**Table 1:** Group 1: Patients with SMA 5q type 1 who only used nusinersen

Patient	Sex	Subtype	*SMN2* copy	Age of disease onset / Age of onset of nusinersen (in months)	Total follow-up (in months)	Maximum score achieved on CHOP INTEND	CHOP INTEND change	Ventilatory support at baseline/at the end	Daily ventilation time at baseline/at the end (in hours)	Feeding route at baseline/at the end	Motor milestones achieved
1	F	C	unknown	3/115	18	19	16	IMV/IMV	24/24	PEG / PEG	None
2	F	C	2	3/12	18	53	12	None/NIV	0/6	Oral / Oral	Sit without support
3	F	C	2	/22	18	26	22	IMV/IMV	13/16	PEG / PEG	Head control
4	M	B	2	2/99	18	6	0	IMV/IMV	24/24	PEG / PEG	None
5	M	C	2	3/20	18	11	1	IMV/IMV	22/23	PEG / PEG	None

Abbreviations: CHOP INTEND, Children's Hospital of Philadelphia Infant Test of Neuromuscular Disorders; F, female; M, male; NIV, noninvasive ventilation; IMV, invasive mechanical ventilation with a tracheostomy; PEG, percutaneous endoscopic gastrostomy.

Comparison with other real-world studies of patients taking only nusinersen reveals that our average increase in the CHOP INTEND scale at 18 months (10.2) falls within the range documented by Erdos and Wild (5.48–19.11 over 12–18 months, patients aged from 2 months to 15 years), although lower than that reported by Belančić et al. (18.2–25.7 over 18 months, patients aged from 1 month to 20 years). 31 32

Remarkably, in group 2 ( Table 2 ), all the patients acquired the motor milestone of sitting without support and 40% walking with support, and all reached a score of at least 48 points on the CHOP INTEND scale. As for the respiratory and bulbar part, none of the patients progressed to invasive mechanical ventilation via tracheostomy, 40% maintained oral feeding with solid foods, and 20% could pronounce sentences. Even after the end of the assessment, the patients of group 2 continued to achieve significant gains. This better outcome could be related to the earlier start of their treatment.

**Table 2:** Group 2: Patients with SMA 5q type 1 who switched from nusinersen to onasemnogene abeparvovec

Patient	Sex	Subtype	*SMN2* copy	Age of disease onset / age of onset of nusinersen (in months)	Age of disease onset / age at the application of onasemnogene abeparvovec (in months)	Total follow-up (in months)	Maximum score achieved on CHOP INTEND	CHOP INTEND change	Ventilatory support at baseline/at the end	Daily ventilation time at baseline/at the end (in hours)	Feeding route at baseline/at the end	Motor milestones achieved
1	M	C	3	5/9	5/16	25	64	25	NIV/NIV	10/11	Oral / Oral	Walk with support
2	M	B	2	1/3	1/13	29	53	35	None/NIV	0/10	Oral / PEG	Sit without support
3	F	B	2	2/12	2/18	23	55	27	None/NIV	0/15	Oral / PEG	Sit without support
4	F	B	2	1.5/1 (pre-symptomatic)	1.5/10	16	64	42	None/NIV	0/14	Oral / Oral	Walk with support
5	F	C	2	3/10	3/19	28	48	36	None/NIV	0/18	Oral / PEG	Sit without support

Abbreviations: CHOP INTEND, Children's Hospital of Philadelphia Infant Test of Neuromuscular Disorders; F, female; M, male; NIV, noninvasive ventilation; PEG, percutaneous endoscopic gastrostomy.

When comparing the motor response with the ventilatory and bulbar response in group 2, we noticed that the latter was not as significant as the first, which is justified by the fact that disease-modifying therapies can lead to motor improvement regardless of whether there is a response in the remaining parameters. 33

**Figure 5:**
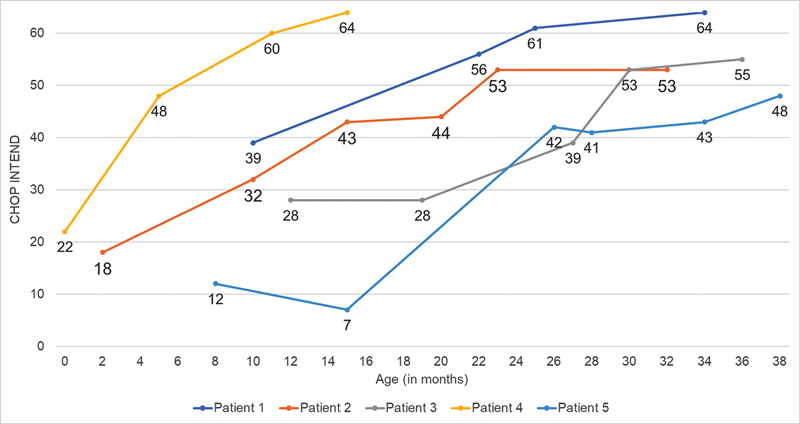
Evolution of the score on the Children's Hospital of Philadelphia Infant Test of Neuromuscular Disorders scale in patients who switched from nusinersen to onasemnogene abeparvovec (group 2).

Among the patients in group 2, only 2 were administered OA before the age of 12 months and both reached the maximum CHOP INTEND scores recorded at the end of the follow-up period ( Figure 5 ). In addition, they were able to walk with support and eat solid foods orally. It should be noted that one of these patients was presymptomatic, while the other had 3 copies of *SMN2* .

Regarding real-world studies of OA usage (with or without prior use of nusinersen), our average 18-month CHOP INTEND score increase (33 points) aligns closely with those reported by Al-Zaidy et al. (an average 28.3-point increase over 24 months) and Stettner et al. (an average 28.1-point increase over 12 months), with all second-group patients reaching motor milestones in these studies. 34 35 However, when comparing with the Brazilian cohort by Mendonça et al., we did not observe an improvement in the ventilatory and bulbar pattern of our patients. 36

Group 2 had a longer follow-up period compared with group 1, due to transitioning from nusinersen to OA and initiating a new 18-month observation period. This difference in observation time may affect the results of group 1, despite most of them (80%) having started treatment using invasive mechanical ventilation with tracheostomy, which can be associated with a worse response. 37 38

Furthermore, the 2 groups are not comparable since the age at initiation of therapy in group 2 was younger than in group 1, and in group 2, there was a presymptomatic patient and another with 3 copies of SMN2, which represents an important bias that may explain the discrepancy related to clinical response between the groups.

To our knowledge, this is the first publication describing a single neuromuscular center patient's response in Rio de Janeiro to OA. Although our study is limited by a small sample size and a short evaluation period, this information is relevant and portrays the effectiveness of disease-modifying therapies, with limited adverse events, leading to a transformation in long-term prognosis, by changing patients' phenotype.
